# Characterization of Mesenchymal Stem Cell Differentiation within Miniaturized 3D Scaffolds through Advanced Microscopy Techniques

**DOI:** 10.3390/ijms21228498

**Published:** 2020-11-11

**Authors:** Valentina Parodi, Emanuela Jacchetti, Arianna Bresci, Benedetta Talone, Carlo M. Valensise, Roberto Osellame, Giulio Cerullo, Dario Polli, Manuela T. Raimondi

**Affiliations:** 1 Department of Chemistry, Materials and Chemical Engineering «G. Natta», Politecnico di Milano, 20133 Milano, Italy; emanuela.jacchetti@polimi.it (E.J.); arianna.bresci@polimi.it (A.B.); manuela.raimondi@polimi.it (M.T.R.); 2 Department of Physics, Politecnico di Milano, 20133 Milano, Italy; benedetta.talone@polimi.it (B.T.); carlomichele.valensise@polimi.it (C.M.V.); roberto.osellame@cnr.it (R.O.); giulio.cerullo@polimi.it (G.C.); dario.polli@polimi.it (D.P.); 3 Istituto di Fotonica e Nanotecnologie (IFN), Consiglio Nazionale delle Ricerche (CNR), 20133 Milano, Italy

**Keywords:** nonlinear microscopy, stem cell differentiation, 3D culture, CARS, SHG

## Abstract

Three-dimensional culture systems and suitable substrates topographies demonstrated to drive stem cell fate in vitro by mechanical conditioning. For example, the Nichoid 3D scaffold remodels stem cells and shapes nuclei, thus promoting stem cell expansion and stemness maintenance. However, the mechanisms involved in force transmission and in biochemical signaling at the basis of fate determination are not yet clear. Among the available investigation systems, confocal fluorescence microscopy using fluorescent dyes enables the observation of cell function and shape at the subcellular scale in vital and fixed conditions. Contrarily, nonlinear optical microscopy techniques, which exploit multi-photon processes, allow to study cell behavior in vital and unlabeled conditions. We apply confocal fluorescence microscopy, coherent anti-Stokes Raman scattering (CARS), and second harmonic generation (SHG) microscopy to characterize the phenotypic expression of mesenchymal stem cells (MSCs) towards adipogenic and chondrogenic differentiation inside Nichoid scaffolds, in terms of nuclear morphology and specific phenotypic products, by comparing these techniques. We demonstrate that the Nichoid maintains a rounded nuclei during expansion and differentiation, promoting MSCs adipogenic differentiation while inhibiting chondrogenesis. We show that CARS and SHG techniques are suitable for specific estimation of the lipid and collagenous content, thus overcoming the limitations of using unspecific fluorescent probes.

## 1. Introduction

### 1.1. Mechano-Regulation of Stem Cells In Vitro by Means of Three-Dimensional Culture Systems

Mesenchymal stem cells (MSCs) are largely used in regenerative medicine thanks to their location in several tissues (i.e., adipose tissue and bone marrow), to their multilineage potential and immunomodulatory effect [1,2]. MSCs in their natural microenvironment reside in the stem niche, which promotes homing, cell renewal, and differentiation through the combination of several biophysical stimuli [3,4]. Replicating these conditions in vitro is crucial for the growth and proliferation of MSCs for stemness maintenance. Traditional culture systems for MSC growth in vitro lack the ability to replicate the in vivo conditions, so that cells tend to differentiate in few passages. The stemness capability is typically maintained through genetic manipulations such as pluripotency induction [5], addition of feeder layers, for example mouse embryonic fibroblasts [6] and chemical xenogeneic factors, such as the leukemia inhibitory factor [7,8]. Since these solutions alter the cell biology raising risks that affect the employability of the cells as direct therapeutic product, alternatives to maintain cellular stemness by the exploitation of cell mechanical conditioning are intensively investigated. In fact, cell mechanics controls several fundamental aspects of cell response; for example the cytoskeleton supports the cell structure, but it also guides cell motility, viscosity and elasticity and, if altered, it can promote pathological conditions such as sickle anemia in red blood cells, tumor progression and laminopathies [9]. Several studies demonstrated the role of the cytoskeleton in driving stem cell fate [10,11,12] by so-called mechanotransduction process, translating mechanical stimuli into biochemical signals: for example, a force transmission between the external and the internal cellular compartments alters cell shape and, thus, the import of gene-regulating transcription factors that, in turn, determine gene expression [13,14]. The research in this novel field focused on the development of engineered systems capable to control cell stemness in vitro through mechanical conditioning [15]. For example, Wei and colleagues, [16] in their study, demonstrated the possibility to maintain embryonic stem cell pluripotency, also in long-time culture, exclusively by mimicking in vitro the spatial configuration of cells in their native environment by the use of 3D scaffolds made of collagen, chitosan and polylactic-glycolic acid. Similarly, Bao et al. [17] studied the effect of scaffold stiffness, cellular confinement and geometrical cues on stem cell morphology and growth. In their study they demonstrated how important is the choice of the culture substrates within stem cell manipulation in terms of cytoskeletal reconfiguration and focal adhesions development [18]. These studies highlight the need for label-free and non-destructive methods for MSCs phenotypic characterization in the field of tissue engineering.

### 1.2. Non-Invasive Label-Free Microscopy Techniques for Stem Cell Characterization

Several biochemical techniques are conventionally used to identify markers of cell differentiation, such as real-time polymerase chain reaction (RT-PCR), DNA sequencing, transcriptome analyses, and protein content (Western blotting) and cyto/histopathological assays. Despite their high information content, these techniques require invasive and, often, destructive treatments like fixation, permeabilization, and extraction of lysates. Optical techniques, on the other hand, are non-destructive and enable three-dimensional observations of viable cells and the study of their functional and morphological properties. Confocal fluorescence is the gold standard in optical microscopy, widely employed in biological laboratories, relying on a wide variety of dyes for live measurements and labelled antibodies for immunofluorescence studies. However, the addition of fluorescent labels can in some cases affect the biochemical phenomenon of interest. Label-free functional biological assessments are useful to overcome these limitations. Nonlinear optical (NLO) microscopy comprises several modalities such as: two-photon excited fluorescence (TPEF) [19], second- and third- harmonic generation (SHG and THG) [20,21] and coherent Raman scattering microscopy (coherent anti-Stokes Raman scattering—CARS—and stimulated Raman scattering—SRS) [22]. By using these techniques, it is possible to investigate unlabeled and vital cells exploiting their intrinsic properties, while irradiated with an ultrashort pulsed laser source. Hence, the autofluorescence of endogenous molecules, the non-centrosymmetric nature of fibrillar proteins, the refractive index mismatch between interfaces of heterogenous materials and the resonant frequencies of the main biochemical bonds can be analyzed, respectively, for each of the above-mentioned techniques [23,24]. In the study of Lee and colleagues [25], MSCs were cultured with differentiation (adipogenic and osteogenic) and expansion culture media and then investigated by broadband CARS to assess the expression of differentiation markers. Spectral analyses and image reconstructions allowed identifying whether stem cells were committed towards their differentiation lineage or not. In fact, a specific spatial distribution of CARS signal at the corresponding resonance frequency was obtained for nuclear and cytosolic domains, while lipid droplets, and mineral deposits were identified for adipogenesis and osteogenesis, respectively. Considering tissue engineering applications, Mortati et al. [26] compared the collagen production, over time, of human adult corneal fibroblasts with MSCs seeded on a 3D fibrin hydrogel scaffold using the combination of CARS and SHG microscopy. Similarly, Lee et al. [27] exploited TPEF and SHG to assess the differentiation capability of MSCs in contact with a 3D polyglycolic acid construct exposed to chondrogenic medium. Hence, quantitative, and morphological estimations of extracellular matrix formation over days suggested that these non-invasive techniques could be employed for real-time monitoring of the matrix production process.

In this work, we study MSCs grown in an innovative 3D scaffold, named Nichoid, designed by our group [28,29,30] primarily to investigate the correlated effects of mechanical stimuli on nuclear membrane permeability to transcription factors involved in stem cell differentiation. The Nichoid remodels the cytoskeleton and conditions cells to maintain a more spherical shape of the nuclei during proliferation with respect to cells grown on flat substrates [28]. Furthermore, we demonstrated that this scaffold maintains MSCs multipotency in vitro [31,32,33]. The architecture of the Nichoids allows optical inspection being composed of micrometric pores (tens of microns in size) and has a thickness of only 30 μm [24]. Here, MSCs were studied at different time points during adipogenic and chondrogenic differentiation inside Nichoids, to assess the effect of the 3D culture on cell morphology and nuclear shape in long-term culture. Then, a comparison between confocal fluorescence microscopy and label-free NLO multimodal microscopy techniques was provided. We employed a nonlinear multi-photon transmission inverted microscope customized by Crisafi and colleagues [34] which is capable to perform 3D sectioning and sequential TPEF/SHG imaging as well as CARS/SRS imaging in the C-H stretching region of the Raman shift. We studied MSCs in live and unlabeled conditions focusing on the CARS and SHG modalities. Our results indicate that the use of non-invasive multimodal NLO microscopy is a valid alternative for MSCs characterization in vitro in vital conditions in a 3D culture microenvironment, an important step forward in view of preclinical and clinical assessments in the context of cell and tissue engineering strategies.

## 2. Results

### 2.1. Characterization of MSCs Differentiation: F-Actin Organization and Nuclear Shape

**Figure 1:**
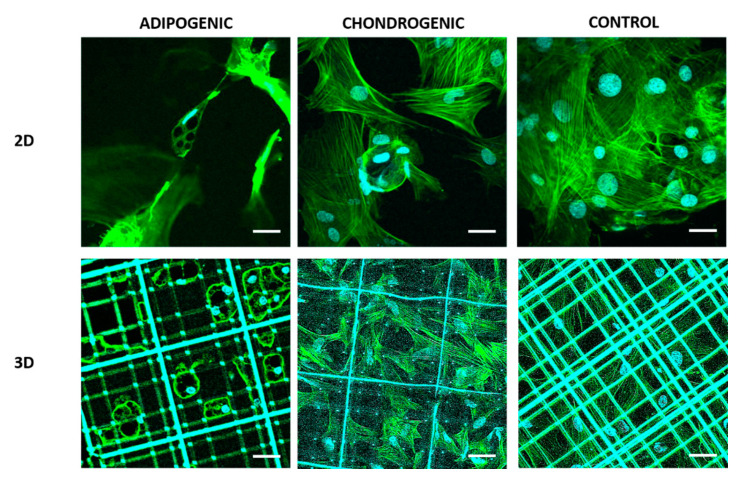
Immunofluorescence images of mesenchymal stem cells (MSCs) at 21 days of culture on flat substrates (**2D**) and on Nichoid scaffolds (**3D**). In green F-actin stained by phalloidin-FITC and in cyan, cell nuclei marked with Hoechst 33342. Scale bars 30 μm.

To investigate the effect of the Nichoid scaffold on MSCs morphology during adipogenic and chondrogenic differentiation, fluorescence analyses were performed since the scaffold allows optical inspections. Figure 1 shows the cell cytoskeletal and nuclear organization highlighted by phalloidin-FITC F-actin staining (in green) and Hoechst 33342 nuclear marker (in cyan) of MSCs exposed to adipogenic, chondrogenic, and basal culture media both inside Nichoids (3D) and on flat glass samples (2D). From a qualitative observation of the acquired images collected through confocal microscopy, the amount of actin fibers inside MSCs involved in adipogenic differentiation appeared to be confined at the cell periphery especially in cells inside Nichoids. Instead, during chondrogenesis, cell morphology on flat substrates appeared from the condensation of cells arranged in a shell structure, while inside Nichoids cells appeared to be stretched in a stellate isotropic configuration characteristic of undifferentiated MSCs. Finally, spread MSCs grown with basal medium (control) for 21 days proliferated, reaching a high population expressing high level of F-actin with respect to cells inside Nichoids, as expected.

**Figure 2:**
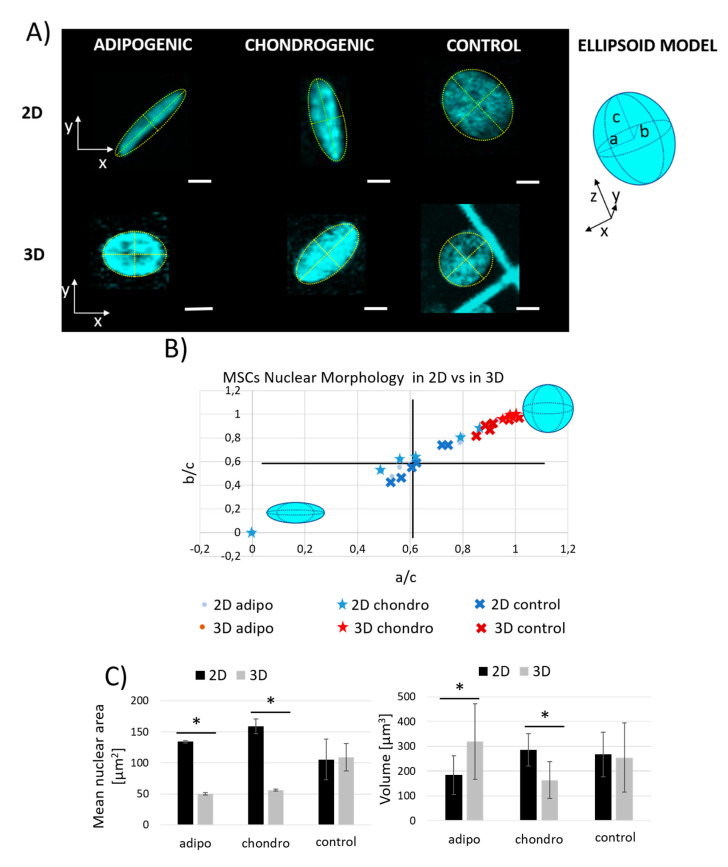
MSCs nuclear morphology assessment inside Nichoids (3D) and on controls (2D) after 21 days of culture with adipogenic, chondrogenic, and basal media. (**A**) Fluorescence images of single cell nuclei stained with Hoechst 33342 obtained by confocal fluorescence microscopy in the different culture conditions and schematic representation of the ellipsoid model of the MSCs nucleus with evidenced semi-axes: *a*, major, *b*, minor and *c*, vertical. Scale bars 5 μm. (**B**) Scatter plot representing the nuclei of differentiated and control cells on 3D Nichoids and on flat glass substrates, in order to compare their nuclear morphologies by means of the relationship on the ratio between the minor (*b*) axis with respect to the vertical (*c*) semi-axis and the ratio between the major (*a*) with respect to the vertical (*c*) semi-axis, respectively. These calculations allowed to investigate the effect of the Nichoid on the nuclear shape in terms of nuclear flatness and elongation. All the parameters considered were measured by Fiji-ImageJ Measure tool for ellipsoid fit, as described in Materials and Methods. (**C**) On the left: quantification of the mean nuclear area of nuclei measured using the maximum projection of all the acquired nuclear planes; on the right, mean nuclear volume, calculated as described in Materials and Methods. * *p* < 0.05.

Focusing the attention on the nuclear shape, evident morphological variations appeared between cells inside Nichoids and cells on controls (Figure 2A). As previously demonstrated [28], MSCs nuclei reconstructed from sequential vertical images can be modeled with an ellipsoid with horizontal major and minor semi-axes, *a* and *b*, and with a vertical semi-axis, *c*. The effect of the Nichoid scaffold on MSCs nuclei resulted in a more compact nuclear shape with a tendency to induce a spherical morphology since the ratio between the minor (*b*) and the vertical (*c*) semi-axes and the major (*a*) and the vertical (*c*) semi-axes is higher than 0.5 (Figure 2B). Contrarily, the behavior of nuclei on flat controls is more heterogeneous. From the analysis of the mean nuclear cross-sectional area, considering the area obtained by measuring the maximum projection of each nuclei, we found that cell nuclei on flat substrates were bigger than the corresponding ones inside the Nichoids for adipogenic and chondrogenic conditioning, while no statistical differences were measured on cells grown with basal medium. In particular, the area of cells exposed to adipogenic and chondrogenic differentiation media inside Nichoids was similar and three times lower with respect to their relative flat counterparts and two-times lower with respect to cells exposed to basal medium. Finally, considering the volumetric distribution of the nuclei, especially inside Nichoids, the average volume of the nuclei was calculated, as indicated in Materials and Methods section, and compared among the cases. The smallest nuclear volume was calculated for chondrogenesis of MSCs inside the Nichoids, while the biggest one was obtained for adipogenesis again inside the Nichoids. No statistical variations were obtained for cells during proliferation in both culture conditions (Figure 2C).

### 2.2. MSCs Differentiation towards Adipogenic Lineage

**Figure 3:**
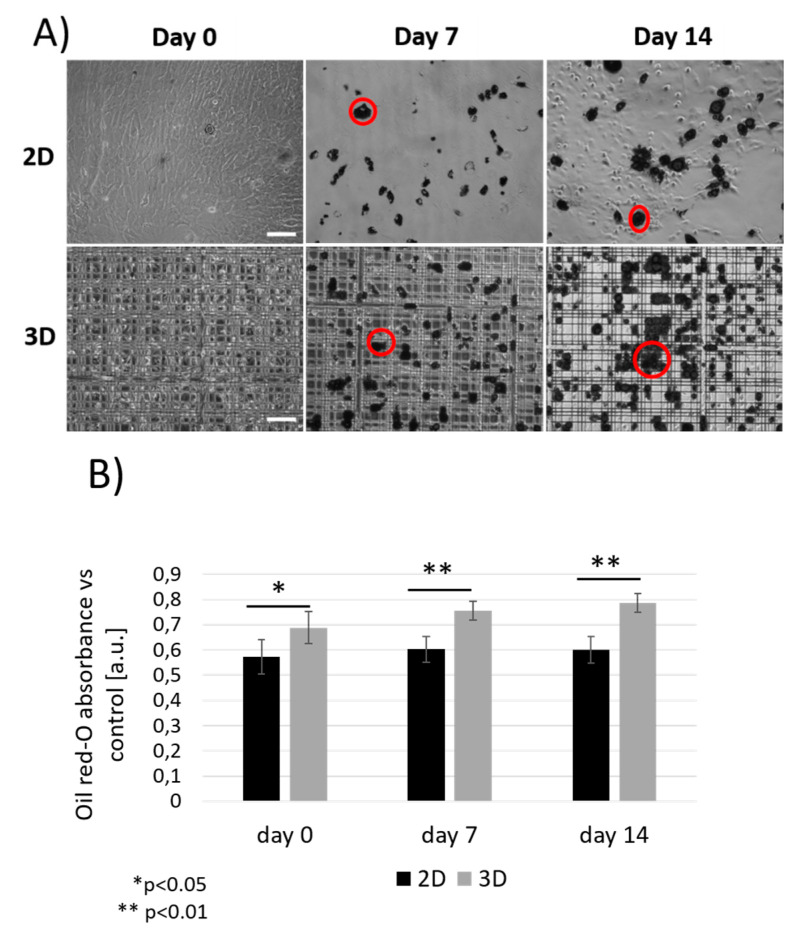
Oil red-O staining of MSCs cultured with adipogenic differentiation media both inside Nichoids (3D) and on glass substrates (2D). (**A**) Brightfield images of differentiated cells taken after 24 h, 7 and 14 days from the beginning of the experiment, in which lipids appeared in dense dark aggregates. Examples of lipid vesicles are highlighted by red circles. Scale bar 100 μm. (**B**) Oil red-O absorbance measured at 490 nm at each time point for both 2D and 3D culture configurations. Data were normalized with respect to values from pure oil red-O and undifferentiated controls. ** *p* < 0.01, * *p* < 0.05 from pair wise comparison.

Adipogenic differentiation of MSCs in vitro was firstly evaluated by the conventional approach with oil red-O staining. In Figure 3A, MSCs were imaged at day 0, 7 and 14 from the initial exposure to adipogenic factors both inside Nichoids (3D) and on control flat samples (2D). Lipid vesicles, positive to oil red-O, appeared as dense red droplets, here shown in black. Adipocytes were more present inside Nichoids with respect to flat substrates both at day 7 and day 14, as indicated by the analysis of the oil red-O absorbance measured at 490 nm after the extraction of the stain from each sample. As shown in Figure 3B, the MSCs differentiation level is 30% larger at day 7 inside Nichoids with respect to controls, and just 35% larger at day 14. These results suggest that the spatial configuration of cells inside Nichoids combined with the stiffness of the scaffold boost adipogenesis once the process has started.

**Figure 4:**
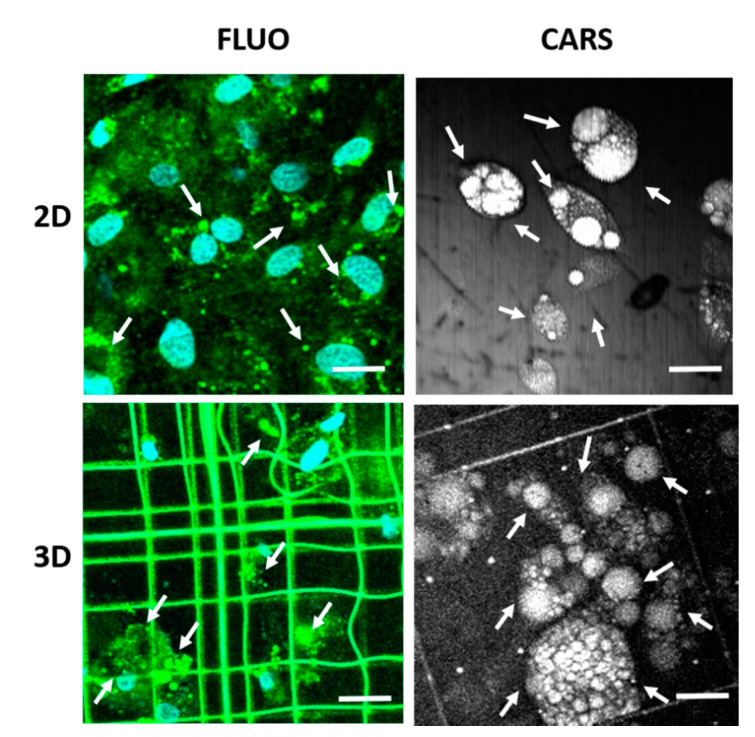
Left: day 14 of MSCs adipogenic differentiation fluorescently labeled, with Hoechst 33342 for nuclei, cyan, and DiO-lipophilic dye, green, for lipids observed through confocal fluorescence microscopy, 512 × 512 pixels. Right: vital and label free imaging obtained through coherent anti-Stokes Raman scattering (CARS) microscopy: 200 × 200 pixels, measured at 2845 cm^−1^. Lipid vesicles are indicated by white arrows. Scale bars 25 μm.

Since the oil red-O assay required invasive and destructive treatments of the biological sample, a second analysis based on vital microscopy for the quantification of lipid production was performed. To understand the level of cell commitment, two imaging techniques were used: CARS microscopy and confocal fluorescence microscopy. Vital differentiated MSCs were imaged through CARS microscopy by setting the frequency of resonance corresponding to 2845 cm^−1^, specific for lipids, to observe vesicles content in both Nichoids and controls at day 7 and 14. Then, samples were stained with Hoechst 33342 to identify nuclei and with a lipophilic tracer (DiO), a long-chain dialkylcarbocyanine commonly used to stain lipids in live cells, for fluorescence confocal microscopy (Figure 4). CARS imaging revealed lipid signal in correspondence of the vesicles, allowing the precise reconstruction of their volumetric distribution both inside Nichoids and on controls.

**Figure 5:**
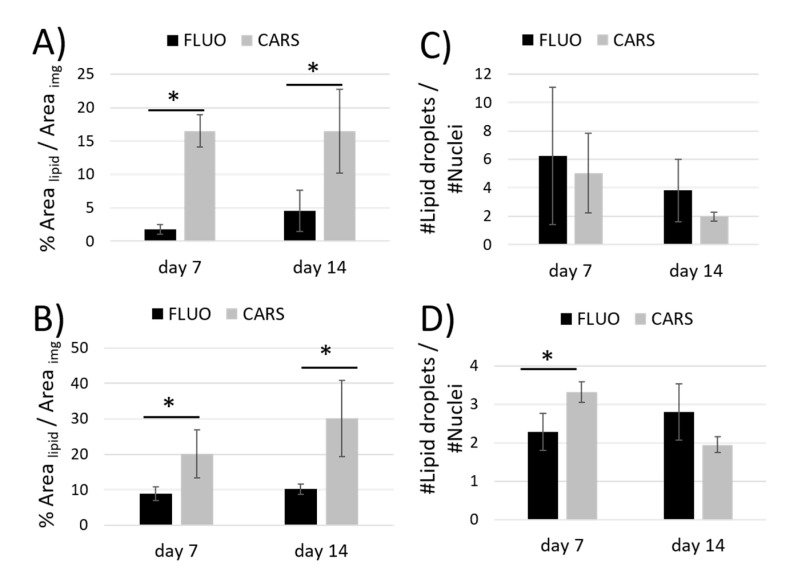
In the graphs, a comparison between confocal fluorescence microscopy and CARS microscopy to assess the lipid amount by calculating the ratio between the area occupied by lipids and the area of the image (**A**,**B**) and the ratio between the number of nuclei and the number of droplets in both culture configurations (**C**,**D**). * *p* < 0.05.

Conversely, the amount of lipid droplets visualized through fluorescence confocal microscopy following DiO staining was overestimated due to the low specificity of the dye. The comparison of the results of the two imaging techniques is shown in Figure 5. The level of differentiation of MSCs was obtained from indirect analysis of the area covered by lipids with respect to the area of the image (Figure 5A,B). The results obtained with both techniques confirmed, as expected, that the level of cell differentiation inside Nichoids is higher than on flat substrates at each time point. Moreover, with these experiments we demonstrated that CARS technique is more sensitive for lipid vesicles quantification. In fact, the estimation of the percentage of lipid areas with respect to the total surface of the image obtained through CARS was higher if compared to fluorescence microscopy in each sample investigated. As shown in Figure 5A, in the case of flat substrates, results obtained with CARS at day 7 and 14 are about 8-folds and 3-folds bigger, while into the Nichoid (Figure 5B) they are 2-folds and 3-folds at day 7 and 14, respectively. Then, the number of lipid vesicles per cell was calculated. This is an interesting data because, coupled with the above information, it allowed us to understand the dynamics of differentiation. The distribution of lipid vesicles per cell was found to be higher at day 7 with respect to day 14 both for the Nichoid and for flat substrates measured with both CARS and fluorescence (Figure 5C,D). Higher values, then, were obtained for fluorescence microscopy and, in particular on flat substrates compared to CARS (Figure 5C). In Figure 5D, fluorescence measurements at day 7 resulted to be 30% lower than the CARS outcome and 20% lower than the fluorescence measurement at day 14. In general, adipocytes tended to produce small vesicles at early stage of differentiation that grew in dimensions, assembling together during the second week, thus resulting in a reduced amount.

**Figure 6:**
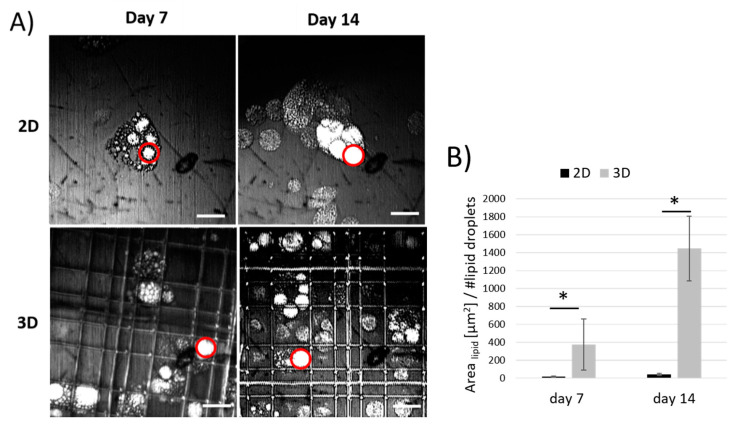
(**A**) Day 7 and 14 analysis of MSCs adipogenic differentiation obtained by vital and-label free CARS microscopy: 140 × 140 μm^2^ at 2845 cm^−1^. Examples of lipid vesicles are indicated by red circles. Scale bars 20 μm. (**B**) Differentiation efficiency represented by the ratio between the total area occupied by lipid droplets in CARS images (140 × 140 μm^2^) and the number of droplets counted in CARS images at day 7 and 14 of differentiation. * *p* < 0.05.

CARS microscopy showed more reliable values in both Nichoids and controls, hence we estimated the size of lipid droplets by analyzing CARS images at day 7 and 14 comparing the two culture configurations (Figure 6A). Figure 6B shows the average area of lipid droplets obtained by calculating the ratio between the total area covered by lipids and the lipid droplets amount from CARS analysis. An overall increase from day 7 to day 14 was found in both Nichoids and controls, but Nichoids offered a higher surface to-volume ratio resulting in larger vesicles with respect to flat substrates of about 2-folds at day 7 and 3-folds at day 14 (Figure 6B). In Supplementary Figure S1 we presented an example of the three-dimensional spatial resolution of the CARS microscope which enabled us to observe the volumetric distribution of lipid droplets produced intracellularly during MSCs adipogenic differentiation. Hence, considering that lipid droplets increase their size while MSCs differentiate inside Nichoids and that a wider area is covered by lipids inside Nichoids with respect to the flat control, we can conclude that Nichoids better promote adipogenesis than flat substrates.

### 2.3. MSCs Chondrogenesis and Collagen Synthesis inside Nichoids

**Figure 7:**
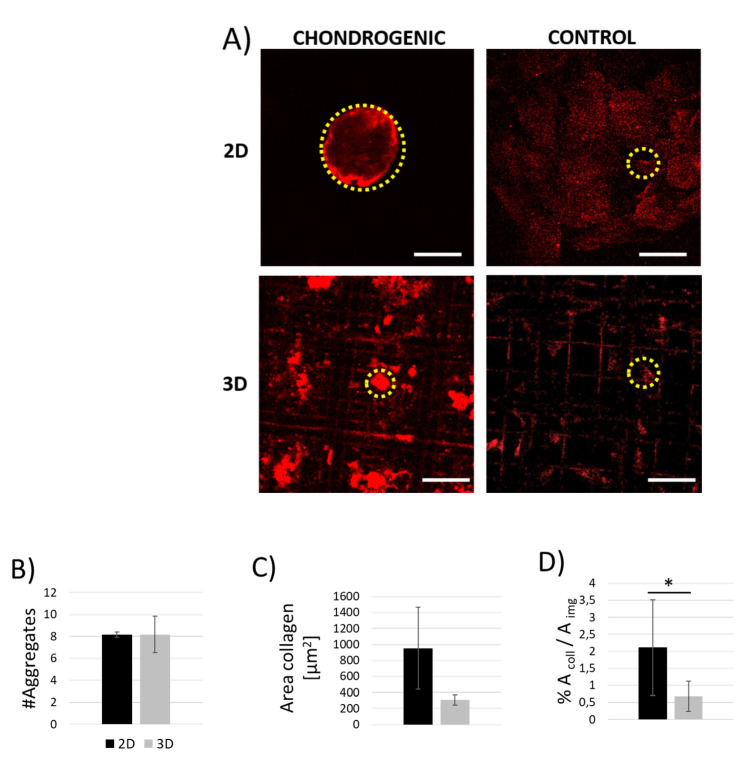
Indirect collagen measurements of MSCs exposed to chondrogenic and basal media at 21 days from the seeding. (**A**) Immunofluorescence images showing in red the signal corresponding to collagen type-I stained with anti-collagen I conjugated to Alexa Fluor 647. Examples of collagen bundles synthetized by MSCs both on glass substrates (2D) and on Nichoid scaffolds (3D), are indicated by yellow circles. Scale bars 50 μm. (**B**–**D**) Collagen-I analysis allowed to quantify the number (**B**) and the average area of collagen aggregates (**C**), and the average total area of collagen produced in each of the cases considered with respect to the area of the image (**D**). * *p* < 0.05.

MSCs differentiation towards chondrogenic lineage both in Nichoid scaffolds and in flat substrates was studied initially through confocal fluorescence microscopy to observe the expression of collagen-I, the main component of the pre-cartilaginous matrix in the early stage of MSCs differentiation [35]. The level of extracellular matrix synthesis and, thus the degree of MSCs differentiation, was obtained by comparing MSCs cultured for three consecutive weeks with chondrogenic medium and with a basal culture medium (control) (Figure 7A). By analyzing the fluorescence images acquired with confocal fluorescence microscopy, it was possible to observe whether the collagen was produced and whether it remained inside the cells. MSCs exposed to chondrogenic medium tended to produce collagen aggregates in dense and spherical masses on flat substrate while small and non-regular deposits were observed inside Nichoids, as shown in Figure 7A. Contrarily, MSCs culture with basal medium for 3 weeks reached confluence on flat substrates (2D) creating highly packed dense colonies and synthesizing collagen-I with heterogenous bundles not ease to detect and estimate. Instead, cells inside Nichoids presented the collagen-I molecule in pre-polymerization phase inside the cells. To evaluate the chondrogenesis kinetics on the different substrates, the number of collagen aggregates per images measured was calculated. Results were constant in the case of induced chondrogenesis both in 3D and in 2D culture (Figure 7B). Considering the average area estimated per aggregates (Figure 7C), chondrogenesis promoted the formation of larger aggregates on flat substrates with respect to Nichoids, since cells were constrained within small surfaces, even if no statistically significant differences were measured. Finally, the mean total area covered by the collagen was measured as described in Methods. In Figure 7D the highest percentage of collagenous area belongs to MSCs grown on flat substrates with chondrogenic medium (2%), as expected. Inside Nichoids collagenous deposits occupied 0.6% of the area of the image in the case of induced chondrogenesis, meaning that only a few intracellular collagenous spots were synthesized.

**Figure 8:**
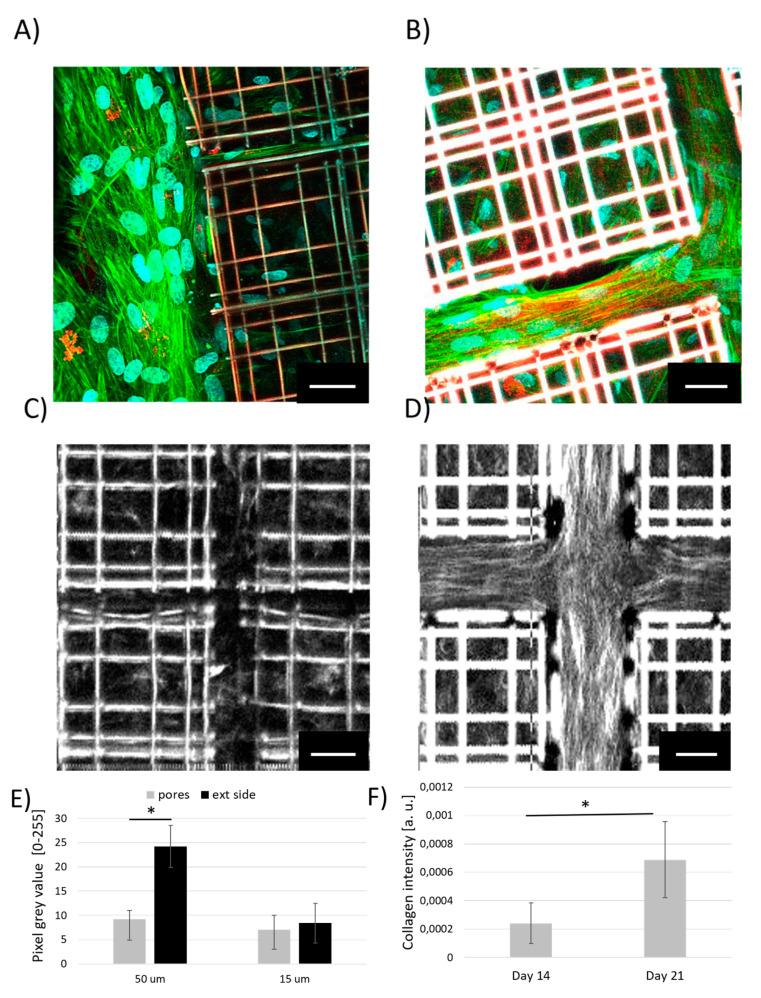
Chondrogenesis of MSCs inside Nichoids. (**A**,**B**) Immunofluorescence images, 512 × 512 pixels, of differentiated MSCs inside Nichoids with standard (**A**) and revised geometries (**B**): collagen appeared in red, F-actin in green and nuclei in cyan. (**C**,**D**) Second harmonic generation (SHG) microscopy of vital and unlabeled cells after three weeks of exposure to differentiation medium inside standard Nichoids (**C**) and in Nichoids with more distant matrixes (50 μm) (**D**), 200 × 200 pixels. (**E**) Collagen-SHG intensity signal measured both inside pores and outside the scaffold in the two Nichoid geometrical configurations. (**F**) Collagen-SHG intensity signal measured in the new geometrical configuration of the scaffold at day 14 and 21 from the beginning of the differentiation. * *p* < 0.05. Scale bars 20 μm.

Given the low amount of extracellular matrix visualized and measured in terms of collagen deposition during MSCs chondrogenic expression in 3D Nichoids, a new initial cell density was set, 50,000 cells/cm^2^, and a revised geometrical configuration of the Nichoid with larger distance among matrixes (50 μm) was employed (Figure 8A–D). To establish whether the geometrical cues were capable to drive MSCs differentiation, a comparison between standard and revised scaffold patterning was provided firstly observing immunofluorescence images of collagen-I in both scaffold geometries (Figure 8A,B), then through SHG microscopy of the collagen fibrils (Figure 8C,D). SHG is capable to reveal the non-centrosymmetric structure of collagen fibrils (polarization-dependent signal) in unlabeled and vital state, visualizing orientated molecules, as clearly visible in Supplementary Figure S2, where vertically polarized light enhances the SHG signal generation of vertically distributed collagen fibrils, while positioning a λ/4 waveplate upwards with respect the focalization objective, the SHG signal appears homogenous in both vertical and horizontal fibers.

From a qualitative comparison of immunofluorescence images of the two Nichoid versions involved in MSCs chondrogenic induction, a confirmation of the collagen presence through antibody-specific staining of collagen type-I (red) was provided in addition to nuclear (Hoechst 33342) and F-actin (phalloidin-FITC) staining (Figure 8A,B). Highly populated Nichoid with a standard geometry showed that cells tended to differentiate better outside the scaffolds with respect to the internal part, and, if compared to more a distanced Nichoid configuration, chondrogenesis was observed more among the scaffold matrixes. Figure 8B suggested that highly oriented and elongated cells experienced anisotropic stresses in the free areas of more distanced Nichoids thus being stimulated to develop fibrillar collagen better than cells exposed to isotropic tensional states inside Nichoids. Hence, label-free SHG observations enabled us to quantify the collagen content in the absence of exogenous molecules. By measuring the intensity of the SHG signal obtained by vertically polarized light, we found low levels of collagen signal both inside and outside standard samples of Nichoids (Figure 8C) after 21 days of differentiation, since the nature of collagen deposits is a little less fibrous than in the larger scaffold, thus being poorly detectable by SHG. Contrarily, a larger scaffold configuration favored the development of collagen fibrils especially located among the matrixes (Figure 8D). Interestingly, SHG revealed a preferential direction of fibrils development along the external walls and corners in the new Nichoid configuration, as visible in Figure 8D. Moreover, SHG signal intensity measurements were found to be 2 times more intense on the outside of the scaffold (ext side) than on the inside of the pores of the Nichoids with a revised geometry, while no significant statistical differences were measured between collagen locations of standard Nichoids (15 μm) (Figure 8E). Then collagen formation inside the new version of the scaffold was observed after two weeks and three weeks of differentiation. In the graph in Figure 8F, the collagen average signal intensity normalized with respect to the area of the image was found to increase over time, as expected. In fact, collagen intensity increased 2.5-folds at day 21 with respect to day 14, suggesting that the production process mainly develops during the third week of differentiation.

## 3. Discussion

### 3.1. Characterization of MSCs Differentiation

F-actin organization and nuclear shape. In this study an investigation of the effect of Nichoids on MSCs during long-term differentiation was performed by optical techniques comparing fluorescence microscopy with label-free and vital nonlinear optical microscopy. Adipogenesis and chondrogenesis of MSCs were considered since tissue characteristics are slightly different in stiffness and genesis [36] and several protocols for in vitro differentiation are available and easy to replicate [30,37]. First, a qualitative observation of MSCs morphology and spatial configuration (Figure 1), combined with a quantitative study of their nuclear shape via confocal fluorescence inspections (Figure 2) was performed in order to assess if evident cellular remodeling arose in cells exposed to chemical differentiation factors with respect to cells exposed to basal growth media inside Nichoids. MSCs during adipogenic differentiation presented a distribution of F-actin close to the cell periphery limited by the presence of lipid vesicles which grew intracellularly occupying the major cellular volume and transforming the cell as a store of triglycerides. In this condition, the presence of the vesicles limits the cellular organelles and the nucleus to the boundaries, since the main cellular function is to synthesize and to accumulate lipids, thus losing cellular motility and contractility [38,39]. Chen et al. [40], in their work, demonstrated by genetic assays and immunostaining that during adipogenic differentiation, a depolymerization of actin fibrils occurs. This behavior was identified in MSCs differentiated both inside and outside the Nichoid except for the nuclear shape that resulted more spherical in 3D with respect to the 2D condition. On flat culture substrates, in fact, it has been demonstrated by Stachecka and colleagues [39] that the variations of cell nucleus during adipogenesis reduced the expression of nuclear lamin A/C, a protein that maintains the nuclear structure, limiting its presence to the rim rather than to the whole nucleus after a week of differentiation, thus favoring nuclear softening and plasticity. In our study, the 3D culture environment offered by the Nichoid scaffold promoted isotropic tensional states thus maintaining a spherical nuclear configuration. Qualitatively, no evident variations in cytoskeletal actin were observed during chondrogenic differentiations inside Nichoids if compared to cells grown with basal medium in 3D conditions. On the contrary, chondrogenic cells on 2D controls showed a particular arrangement of actin that resulted laterally stretched around a bulky sphere, completely far from adherent MSCs exposed to basal medium. Dense colonies of cells are necessary to reach a proper density for chondrogenic differentiation in vitro, thus affecting cellular and nuclear morphology [41]. In fact, the chondrogenic culture which led to spherical constructs on flat substrates, showed a ring of actin and nuclei surrounding an extracellular product of cells (collagen) thus resulting laterally stretched. This behavior was not present during chondrogenesis inside Nichoids since a limited cell-cell contact and a 3D stellate distribution experienced by each cell delayed or inhibited the phenotypic expression. Tigli and colleagues [42] demonstrated that cell cultured on chitosan and on silk fibroin scaffolds enhance cell aggregation and chondrogenic differentiation since cells experienced a more rounded and spherical morphology. This condition is not directly provided inside Nichoids, while it is favored during cell aggregation on flat substrates.

### 3.2. MSCs Differentiation towards Adipogenic Lineage

The analysis of oil red-O absorbance represents the gold standard for the identification of the lipid content in biological tissues. Here, MSCs adipogenic differentiation showed that cells differentiate more inside Nichoids than on controls after 7 and 14 days of induction with respect to flat controls (Figure 3). This suggested that the stiffness of the synthetic micro-structured niche boosts adipogenesis and thus better resembles the condition of a soft tissue with respect to flat controls. Then, a demonstration of the efficacy of CARS microscopy for the analysis of lipid droplets in terms of size and volumetric distribution with respect to vital fluorescence microscopy, especially in 3D sectioning, was provided (Figure 4 and Figure 5). Here, the use of stains resulted in an overestimation of the lipid content since a non-specific binding of the dye occurred. CARS tuned to the resonance frequency of the C-H bonds at the wavenumber 2845 cm^−1^ enabled a time-evolution study of adipogenic differentiation, replicating the trend of oil red-O analysis in viable and unstained 3D samples. The analysis of CARS images allowed to quantify and measure the size of the lipid vesicles, thus suggesting that these underwent a fusion from the first to the second week of differentiation, becoming larger (Figure 6). Isomäki and colleagues [43] described in their study the methods used to characterize MSCs adipogenic differentiation by comparing gene expression of PPAR-γ, lipoprotein lipase (LPL), and leptin expressed at different stages of differentiation, with oil red-O imaging and label-free CARS microscopy at 2845 cm^−1^. CARS allowed non-invasive and repetitive study of MSCs differentiation without the need for cell fixation and staining, ensuring chemical specificity thanks to the identification of vibrational resonances. Similar outcomes were obtained in the work of Smus and colleagues [44] in which skeletal stem cells were induced to differentiate towards adipocytes on flat substrates. By comparing gene expression assays with measurements from CARS images they confirmed an increase in droplets size and in lipid ratio (between the lipids area and the cell area) over time.

### 3.3. MSCs Chondrogenesis and Collagen Synthesis inside Nichoids

The study of chondrogenic differentiation within Nichoid scaffold through imaging techniques enabled to visualize and estimate the synthesis of extracellular collagenous deposits in 3D and 2D culture configuration. From the analysis of collagen-I imaged through confocal microscopy, a low level of collagen formation was measured within Nichoids with respect to flat controls (Figure 7). In fact, in vitro chondrogenesis required highly packed and dense cultures (micro-masses) to favor cell-cell contacts, adhesions, and growth factors import [45,46]. Chondrogenesis involves several passages followed by morphological variations to develop a mature cartilaginous tissue. Therefore, cell spreading inhibits the condensation process and thus no differentiation occurs [47,48]. Hence, to boost chondrogenesis in 3D, an increased initial cell density and a revised Nichoid geometry with more distanced matrixes were introduced. This analysis was performed via SHG microscopy to detect the signal emitted from collagen fibrils (Figure 8). The SHG signal and, therefore, the images obtained by nonlinear microscopy allowed us to observe the fibrillar nature of the collagen, something that fluorescence microscopy did not show (Figure 8A–D). In fact, collagenous compounds on flat control samples could not be observed with SHG since these samples provided a less organized collagen structure. The collagen content estimated from the SHG images increased drastically especially outside Nichoids where the collagen fibrils developed (Figure 8E). This suggested that the substrate, obtained with a denser and more concentrated number of cells, favored chondrogenesis, resembling better the micro-masses configuration [49,50]. Despite SHG microscopy provided a specific detection of collagen fibrils, this NLO technique suffered from autofluorescent signal of the Nichoid scaffold. Martinez et al. [51] employed CARS and SHG microscopy to study the development of collagenous fibrils from fibroblasts stimulated through cellulose micro-channels. Similar to what we found by enlarging the distance among scaffold matrixes, Martinez demonstrated that collagen deposited in highly oriented manner in the whole volume of the channel without any external intervention except for the mechanical one. Furthermore, the presence of collagen observed by CARS microscopy resulted in a different signal localization with respect to the SHG one, as found in other works [52,53], giving a wider information content with respect to fluorescence microscopy of fixed and stained samples.

## 4. Materials and Methods

### 4.1. Microfabrication of Nichoids

3D Nichoids were fabricated on circular glass coverslips with 150–170 μm thickness and 12-mm diameter (Bio-Optica, Milan, Italy). Nichoids were fabricated in serial blocks constituted by squared matrixes of 25 elements, 450 μm × 450 μm × 30 μm each, to cover a whole circular area of 8 mm in diameter. Glass substrates were previously covered with 30 μL of SZ2080 photoresist by drop-casting and then exposed to localized infrared radiation to polymerize single structures. Nichoid microfabrication through two-photon polymerization was performed as detailed in [54]. To remove the unpolymerized resin, samples were developed in solvent solution and characterized through scanning electron microscopy (Phenom Pro, Phenom World, Eindhoven, The Netherlands) to ensure their integrity. Before cell culture, samples were washed three times with sterile deionized water, then fully covered with ethanol 70% and exposed to ultraviolet (UV) radiation for 30 min. Two types of 3D scaffold configurations were employed: the first, defined standard, presented a distance among Nichoid matrixes of 15 μm, which was increased to 50 μm in the second one, thus leaving more available surface for cells to spread.

### 4.2. Cell Culture and Differentiation

Rat bone marrow-derived MSCs at low passages (maximum passage 2) were initially expanded with standard alpha-MEM phenol red-free culture medium (Pan-Biotech GmbH, Aidenbach, Germany) supplemented with 20% of fetal bovine serum, 1% L-glutamine (2mM), penicillin (10 units/mL), and streptomycin (10 µg/mL) at 37 °C and in 5% CO_2_ (Euroclone, Italy). MSCs were differentiated towards adipogenic and chondrogenic phenotypes by adding a specific cocktail of growth factors to a basal medium composed by Dulbecco’s modified essential medium (DMEM) phenol red-free (Gibco, Life Technologies, Carlsbad, CA, USA) supplemented with 10% of fetal bovine serum, 1% L-glutamine (2mM), 1% penicillin (10 units/mL), and streptomycin (10 µg/mL)).

MSCs adipogenic differentiation was performed by seeding 20,000 cells/cm^2^ on Nichoids and 10,000 cells/cm^2^ on glass flat substrates. Cells were maintained in culture with a medium supplemented with 5 μg/mL human insulin (Sigma-Aldrich St. Louis, MO, USA), 1 μM dexamethasone (Sigma-Aldrich), 0.5 μM 3-isobutyl-1-methylxanthine (Sigma-Aldrich) and 50 μM indomethacin (Sigma-Aldrich) for 72 h and, after, with insulin for 24 h. This procedure was cyclically repeated for the 2 weeks of the experiment [30].

MSCs chondrogenic differentiation was obtained by seeding initially 20,000 cells/cm^2^ increased in a second experiment to 50,000 cells/cm^2^ on Nichoids and 5000 cells/cm^2^ on glass flat substrates. MSCs were fed for 3 weeks with a chondrogenic induction medium made by mixing 100 nM dexamethasone, 10 ng/mL transforming growth factor-β1 (PeproTech, Rock Hill, SC, USA), insulin-transferrin-selenium-G 1X (Thermo Fisher), 50 μg/mL ascorbic acid—2-phosphate to the basal medium [37].

### 4.3. Oil Red-O Assay

To quantify the level of differentiation of MSCs after 0, 7, and 14 days from the beginning of the adipogenic induction, oil red-O staining was performed on 3 Nichoids and 3 glass flat samples exposed to adipogenic medium and on the same number of samples exposed to basal medium as a control. At each time point, 2 Nichoids exposed respectively to adipogenic and basal medium and 2 flat glass substrates, in the same conditions, were fixed and colored with oil red-O assay (Sigma Aldrich) 0.1% in isopropanol 60% for 10 min. Before oil red-O extraction, samples were imaged through brightfield microscopy (Olympus IX70, Olympus, Tokyo, Japan). Then, samples were exposed to isopropanol 100% and 100 μL of the extraction production were collected for triplicate reads in a 96-well plate. The absorbance measurement of the lysate was taken at 490 nm with plate reader (Infinite PRO Monochromator, Plate nanoquant Tecan, Männedorf, Zurich, Switzerland).

### 4.4. Vital Fluorescence Staining for Adipogenic Differentiation

To observe and quantify the lipid content of MSCs differentiated towards adipogenic phenotype, 2 Nichoids and 2 glass flat substrates were analyzed, respectively at day 7 and 14 from the beginning of the differentiation through confocal fluorescence microscopy. Cells were stained with 1 μg/mL of Hoechst 33342 for nuclei identification and 5 μg/mL of lipophilic tracers -DiO (Thermo Fisher Scientific, Monza, Italy) for lipids. Image size was set at 212.13 μm × 212.13 μm (512 × 512 pixels) and multiple scans along the Z direction with 1 μm step were captured to ensure a complete volumetric observation.

### 4.5. CARS Lipid Imaging and Analysis

For vital and label-free 3D nonlinear imaging of MSCs exposed to adipogenic differentiation medium, CARS microscopy was performed on a couple of Nichoids and flat controls. Lipid droplets were imaged at day 7 and 14 both on Nichoids and on flat glass substrates through label-free CARS microscopy at the vibrational Raman frequency of 2845 cm^−1^. Image size was set at 140 μm × 140 μm (200 × 200 pixels) and multi-stacks along Z direction were captured with 2 μm of step for 3D Nichoids. The lipid content was measured by analyzing 3 images per sample type at each time point, by manually drawing a circular region of interest (ROI) through Fiji-ImageJ software (NIH- USA, Bethesda, MD, USA). The area of each ROI was extrapolated through the Measure tool of Fiji-ImageJ. Then, the number of lipid droplets and the total area covered by lipids was obtained by summing the areas of single droplets for each image.

### 4.6. Immunofluorescence

We performed immunofluorescence staining to characterize actin organization and nuclear shape of MSCs during adipogenic and chondrogenic differentiation with respect to cells exposed to basal medium, and to quantify the production of collagen-I during chondrogenesis. Experiments were performed on 6 standard Nichoids, 2 Nichoids with a revised geometry, and 6 flat glass substrates. After 3 weeks of cell culture, samples were washed in phosphate buffered saline solution (PBS, Euroclone, Pero (Milan), Italy) three times, fixed in paraformaldehyde 4% for 10 min, rinsed with PBS, permeabilized with 0.25% Triton X-100 (Sigma Aldrich)-PBS for 15 min at room temperature, and finally processed for indirect immunofluorescence analysis. Cells were first incubated for 3 h with a solution of PBS, 2% bovine serum albumin (BSA, Sigma Aldrich) and tween 0.1% (Sigma-Aldrich), then incubated with mouse monoclonal anti-collagen-I antibody 2 μg/mL (Thermo Fisher Scientific) at 4 °C, overnight. After washing 3 times the samples were incubated for 1h at room temperature with 1 μg/mL secondary antibody (Alexa Fluor, Abcam). After three other washes in PBS, 1 μg/mL phalloidin-FITC (Sigma Aldrich) was added and incubated for 30 min to stain F-actin. After three washes, cell nuclei were stained with a 10-min incubation using 1 μg/mL of Hoechst 33342 (Thermo Fisher Scientific, Monza, Italy) in PBS. Samples were then mounted with Mowiol (Dabco) on standard microscope slides and sealed to undergo fluorescence imaging by the use of confocal microscope (Olympus FluoView FV10i, Olympus, Segrate, Italy). Images were collected with a pinhole aperture of 1.5 Airy unit, over a 212.13 μm × 212.13 μm (512 × 512 pixels) field of view and multiple scans along Z direction with 1 μm step to ensure a full volume observation (40 μm for Nichoids and 20 μm for controls).

### 4.7. Fluorescence Images Analysis

Fluorescence images were manipulated with the open-source software Fiji-ImageJ and the measured data collected and analyzed through the software Excel (Microsoft, Redmond, WA, USA).

1. Characterization of the nuclear shapes. Three images per sample type i.e., adipogenic and chondrogenic differentiation and control cells grown with basal medium respectively in Nichoid and in flat control, were analyzed to extract data on nuclear morphology. The count of the nuclei was obtained by manually drawing a polygonal ROI for each of the nuclei of each image and, to avoid errors, a single stack analysis was perform to separate overlapped nuclei and to better exclude the autofluorescent signal from the scaffold. Considering ten nuclei per image in randomized positions the mean area, the vertical (*c*), the major (*a*) and minor (*b*) semi-axes, the Feret diameter and the nuclear aspect ratio were measured through the Measure tool of Fiji-ImageJ. To calculate the maximum projected area of nuclei per sample type, the mean value among each population was calculated with the relative standard deviation. The nuclear volume was calculated for each nucleus considering the formula of the ellipsoid volume (1).
  *V* = 4 ÷ 3∙ *π*∙ *a* ∙ *b* ∙ *c* (1)
2. Lipid vesicle size and distribution during adipogenic differentiation. To quantify the amount and the size of lipid droplets produced during each time point in both culture conditions, three images per sample were analyzed. Droplets stained with DiO were manually drawn by a circular ROI and extracting the area through the Measure tool of Fiji-ImageJ. Hence, the number of droplets was obtained counting the number of areas drawn and to quantify the total area of lipids, the sum of the areas of single droplets was provided per each image. Then, nuclei stained with Hoechst 33342 were manually counted on single stack of images both on 2D and in Nichoids, to estimate the distribution of droplets per nuclei.

Collagen aggregates quantification during MSCs chondrogenic differentiation. To assess collagen synthesis during chondrogenesis, 3 randomized acquired images per sample were analyzed by drawing manually a polygonal ROI, pixel-by-pixel, on collagen deposits areas both on 2D and 3D samples considering the maximum cross section (z-projection). To evaluate the size of collagen deposits, the percentage of area occupied by the collagen with respect to the area of the image was measured by the Measure tool Area in the Fiji-ImageJ measurement settings. Furthermore, a ratio between the number of nuclei and the number of collagen deposits was calculated and compared in all the cases. Statistical differences among culture configurations and media conditioning were established after normality test and Student *t* test.

### 4.8. SHG Collagen Imaging and Quantification

To establish whether Nichoids inhibited collagen type-I synthesis during MSCs chondrogenic differentiation, high-density Nichoids were studied through SHG microscopy in label-free and vital conditions. Here, a standard Nichoid and a Nichoid with a revised geometry were imaged after 3 weeks of differentiation. Secondly, differentiated samples with the new Nichoid configuration were studied at day 14 and 21 of differentiation. SHG images were captured approximately at 10 μm of vertical distance from the glass (approximately half of the Nichoid height) sized about 200 μm × 200 μm (200 × 200 pixels) with a pixel dwell time of 10 ms. Since SHG microscopy depends on the polarization of the light with respect to the orientation of the molecular dipoles, all the samples analyzed were positioned in the same direction. Five SHG images per sample were then analyzed through Fiji-ImageJ to quantify the amount of collagen produced and to establish the effect of Nichoids with respect to flat areas: the SHG signal intensity was measured and compared with the signal from the internal pores of the scaffolds. Statistical analyses were performed after normality test and student *t* test.

### 4.9. Nonlinear Optical Microscopy

Our nonlinear multimodal microscope capable to perform in parallel CARS/TPEF/SHG and SRS imaging by the use of two near-infrared ultrashort pulsed laser sources was previously described in [34]. This custom-made system allowed to scan the sample area both by moving the translational stage in X and Y (8MTF-102LS05, Standa, Vilnius, Lithuania) or by bi-axial galvanometric mirrors (GVS012, ThorLabs, Newton, NJ, USA). The pump beam centered approximately at 780 nm overlapped temporally and spatially with the Stokes one, tunable between 940 nm and 1020 nm, stimulated the generation of an anti-Stokes emission (CARS) detected by a photomultiplier tube (R3896, Hamamatsu) with a band pass filter at 650 nm with 40-nm bandwidth (FB650-40, ThorLabs) coupled with a set of short-pass filters aimed at eliminating the intense pump pulse and the fluorescence produced. To detect the forward SHG signal of the 780 nm beam on the same branch of the CARS (vertically polarized), the set of filters was changed with a sharp band pass filter at 390/18 nm (FF01-390/18-25, Semrock, Rochester, NY, USA) coupled with a short pass filter at 750 nm (FF01-750/SP-25, Semrock). The customized software was developed with MATLAB (MathWorks) and allowed us to control the hardware, by choosing the size of the scanning area, the vertical thickness and the steps, the wavenumber of the desired Raman shift, the focal point and the acquisition speed, and to collect the data via an analog-to-digital conversion card. The software was also equipped with a spectral scanner, allowing to measure a CARS spectrum if necessary, to establish the frequency of resonance.

## 5. Conclusions

In the field of tissue engineering and regenerative medicine, the use of MSCs is growing thanks to their adhesive, immunoregulatory and stemness properties. Their multilineage potential, combined with a larger availability in adult mammalians with respect to other cell types, makes MSCs a valid candidate for scaffold-based constructs in bioengineering applications [2]. The development of 3D culture systems helped researchers to investigate MSCs behavior in vitro by recreating a more in vivo-like microenvironment with respect to traditional tissue culture plates [16,55]. Furthermore, there is a clear evidence of the relationship between topographical/mechanical cues and MSCs differentiation [11,12]. The 3D Nichoid scaffold shapes the MSCs nucleus to a roundish configuration, since the scaffold’s architecture, made by lattice grids, ensures isotropic tensional states [28,31,56,57]. Then, the total Nichoid thickness of 30 μm, not only enables cells to experience a 3D environment, but also favors non-destructive and vital microscopy investigations. In this paper, we studied MSCs adipogenic and chondrogenic differentiation inside the Nichoid scaffold by comparing confocal fluorescence microscopy with nonlinear optical microscopy techniques, such as CARS and SHG. We demonstrated that the MSCs nuclear shape inside Nichoids remained roundish with respect to cells grown on flat glass substrates, thus suggesting that mechanical conditioning prevails on chemical conditioning in this regard. The use of CARS microscopy enabled us to image and quantify the intracellular lipid vesicles produced during MSCs adipogenesis at different time points, with higher accuracy with respect to the less specific DiO fluorescence staining. The size and number of droplets synthesized was found to increase when cells differentiated inside Nichoids, with respect to flat controls. On the contrary, MSCs chondrogenic differentiation was found to be reduced inside Nichoids and enhanced in the channeled spaces between the scaffold matrixes when studied through SHG and immunofluorescence. To conclude, this paper demonstrates the potentiality of NLO microscopy to study biological specimens in vital and label-free conditions within both 2D and 3D configurations, enabling the characterization of stem cell in vitro differentiation in a non-invasive modality.

## Supplementary Materials

The following are available online at https://www.mdpi.com/1422-0067/21/22/8498/s1.

## Abbreviations

2D	Bi-dimensional
3D	Three-dimensional
BSA	Bovine serum albumin
CARS	Coherent anti-Stokes Raman scattering
DMEM	Dulbecco’s modified essential medium
FITC	Fluorescein isothiocyanate
LPL	Lipoprotein lipase
MSC	Mesenchymal stem cell
NLO	Nonlinear optical
PBS	Phosphate buffered solution
PPAR-γ	Peroxisome proliferator-activated receptor gamma
ROI	Region of interest
SHG	Second harmonic generation
SRS	Stimulated Raman scattering
SZ2080	Silicon-zirconium-20%-80%
TPEF	Two-photon excited fluorescence
UV	Ultraviolet
