# Supplementary material for: Characterization of Mesenchymal Stem Cell Differentiation within Miniaturized 3D Scaffolds through Advanced Microscopy Techniques
Source: Int J Mol Sci. 2020 Nov 11;21(22):8498. doi: 10.3390/ijms21228498 (PMC7696107; doi:10.3390/ijms21228498)
Supplement: Supplementary file 1 [file ijms-21-08498-s001.pdf]

Characterization of Mesenchymal Stem Cell Differentiation within Miniaturized 3D Scaffolds through Advanced Microscopy Techniques – Supplementary Materials

Valentina Parodi , Emanuela Jacchetti, Arianna Bresci, Benedetta Talone , Carlo M. Valensise, Roberto Osellame , Giulio Cerullo, Dario Polli and Manuela T. Raimondi

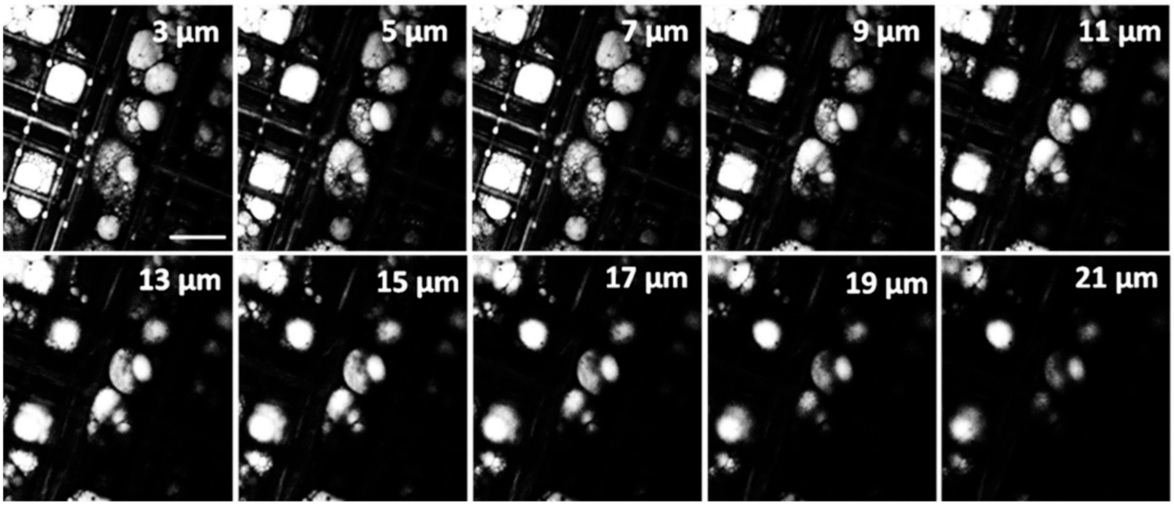

Figure S1. CARS images (140 × 140 µm^2) of lipid droplets at 2845 cm^{-1} obtained by sectioning vertically every 2 µm of 21 µm of thickness the same area of the 3D sample at day 14 from the beginning of the differentiation. Scale bar 15 µm.

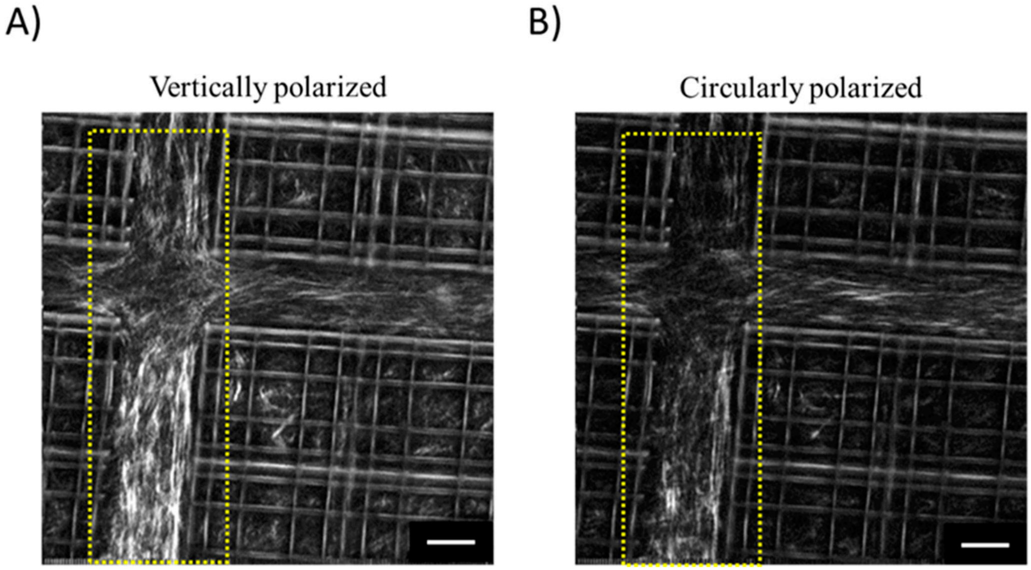

Figure S2. SHG images of the same area of vital and unlabeled MSCs chondrogenesis inside Nichoids at 21 days from the beginning of the differentiation, obtained by irradiating the sample with vertically polarized light (**A**) and with circularly polarized light (**B**) by positioning a λ/4 waveplate upstream

with respect to the focusing objective. Highlighted by yellow region of interest (ROIs) the vertical region in which an evident decrease in signal intensity appeared while using a circular polarizer. Scale bars 30 μm.
